# The dual role of *Escherichia coli* in the course of ulcerative colitis

**DOI:** 10.1186/s12876-016-0540-2

**Published:** 2016-10-10

**Authors:** Magdalena Pilarczyk-Zurek, Magdalena Strus, Pawel Adamski, Piotr B. Heczko

**Affiliations:** 1 Department of Microbiology, Jagiellonian University Medical College, Czysta 18 Street, 31-121 Cracow, Poland; 2 Polish Academy of Sciences, Institute of Nature Conservation, 33 Mickiewicza Avenue, 31-120 Cracow, Poland

**Keywords:** Ulcerative colitis, *Escherichia coli*, Ferrous acquisition, Catalases

## Abstract

**Background:**

This study examines the dual role of *Escherichia coli* in the course of ulcerative colitis (UC). The intestinal microbiota is considered to play an important role in UC pathogenesis, but how *E. coli* contributes to inflammation in UC is still unknown. On the one hand, we demonstrated that there was a significant increase in the number of *E. coli* at the sites of inflammation in patients with UC, which can lead to immune system activation, whilst, on the other hand, *E. coli* may contribute to the resolution of inflammatory reactions since *E. coli* can inhibit hydroxyl radical formation by eliminating substrates of the Fenton reaction, by assimilating ferrous iron (Fe^2+^) and inducing the decomposition of hydrogen peroxide (H_2_O_2_). On this way, *E. coli* may affect the initiation and/or prolongation of remission stages of UC.

**Methods:**

Ten *E. coli* strains were isolated from the colonic mucosa of patients in the acute phase of UC. Using PCR, we examined the presence of genes encoding catalases (*katG* and *katE*) and proteins participating in iron acquisition (*feoB, fepA*, *fhuA*, *fecA*, *iroN*, *fyuA*, and *iutA*) in these *E. coli* strains. To determine if iron ions influence the growth rate of *E. coli* and its ability to decompose H_2_O_2_, we grew *E. coli* in defined culture media without iron (M9(-)) or with ferrous ions (M9(Fe^2+^)). Expression levels of genes encoding catalases were examined by real-time PCR.

**Results:**

All investigated *E. coli* strains had catalase genes (*katG, katE*), genes coding for receptors for Fe^2+^ (*feoB*) and at least one of the genes responsible for iron acquisition related to siderophores (*fepA*, *fhuA*, *fecA*, *iroN*, *fyuA*, *iutA*). *E. coli* cultured in M9(Fe^2+^) grew faster than *E. coli* in M9(-). The presence of Fe^2+^ in the media contributed to the increased rate of H_2_O_2_ decomposition by *E. coli* and induced *katG* gene expression.

**Conclusions:**

*E. coli* eliminates substrates of the Fenton reaction by assimilating Fe^2+^ and biosynthesizing enzymes that catalyze H_2_O_2_ decomposition. Thus, *E. coli* can inhibit hydroxyl radical formation, and affects the initiation and/or prolongation of remission stages of UC.

## Background

Ulcerative colitis (UC) is a chronic non-specific inflammatory disease characterized by inflammation that is limited to the mucosa of the colon and rectum. The characteristic symptoms of UC are bloody diarrhea and abdominal pain. Clinically, the course of UC commonly consists of periods of exacerbated inflammations and remissions. Disease activity is determined on the basis of medical history and endoscopic changes in the colon [1, 2]. Genetic predisposition, disorders of the immune system, environmental factors, and the intestinal microbiota are the primary factors contributing to the etiology of UC [3]. Currently, the intestinal microbiota is considered to play an important role in UC pathogenesis [4, 5]. *E. coli* has been specifically highlighted for its role in the propagation and maintenance of chronic inflammation in UC. The biology of *E. coli* suggests that it may play a double role and show the ability both to increase or decrease gut inflammation. For example, high levels of *E. coli* gut colonization are correlated with high concentrations of its lipopolysaccharide, which activates the host immune system. However, *E. coli* also has features that can promote the resolution of intestinal inflammation [6, 7].

In the exacerbation stage of UC, the action of an increased number of reactive oxygen species (ROS) intensifies necrosis of the intestinal epithelium. One of the most active forms of ROS is the hydroxyl radical (OH^.^), which is a product of the Fenton reaction.

Fenton reaction: Fe^2 +^ + H_2_O_2_ → Fe^3 +^ = OH^‐^ + OH

*E. coli* can eliminate substrates of the Fenton reaction by assimilating ferrous ions and biosynthesizing enzymes that catalyze hydrogen peroxide (H_2_O_2_) decomposition to oxygen and water [8, 9]. *E. coli* uses numerous systems to uptake iron. In the cytoplasmic membrane, the FeoB protein, encoded by *feoB* gene, is the primary transmembrane transporter of Fe^2+^ of *E. coli* [10]. Additionally, *E. coli* excretes siderophores, which are ferric iron (Fe^3+^) chelating compounds. Nearly all *E. coli* strains produce enterobactin, which is one of the most effective siderophores. The synthesis of enterobactin receptor protein is dependent on the gene *fepA*. The receptor proteins for salmochelins (glycosylated forms of enterobactin) are synthesized with *ironN* gene contribution. The *fhuA*, *fecA*, *fyuA*, and *iutA* genes contribute to the biosynthesis of the following siderophores: ferrichromes (hydroxamate), rhizoterins (alpha-hydroxycarboxylates), yersinibactins (phenolate), and aerobactins (mixed-hydroxymate derivatives), respectively [11, 12].

Hydroperoxidases (catalases) limit ROS accumulation and, thus, are an integral component of how bacterial cells respond to oxidative stress. *E. coli* produces two types of hydroperoxidases, catalase/peroxidase I (HPI) and hydroperoxidase II (HPII), encoded by *katG* and *katE*, respectively. HPI and HPII have different structures and kinetic properties. HPI has a minor hydroperoxidase activity in the total activity of catalase, however, it is the most important component of the bacterial resistance to H_2_O_2_. HPI possesses both catalase and peroxidase activity, unlike HPII. The active center of each hydroperoxidase contains a heme system with an iron molecule inside. The presence of iron is essential to the biosynthesis of the active form of both HPI and HPII [13].

We investigated mechanisms by which *E. coli* may influence chronic intestinal inflammation. Specifically, we raised a question whether *E. coli* induces the attenuation of inflammation by eliminating substrates of the Fenton reaction. We aimed to: (1) detect genes that facilitate iron ion acquisition (*feoB*, *fepA*, *fhuA*, *fecA*, *iroN*, *fyuA*, *iutA*), (2) establish whether the growth rate of *E. coli* increases in media supplemented with Fe^2+^, (3) compare catalase activity by assaying the kinetics of H_2_O_2_ decomposition in media with and without Fe^2+^ supplementation, and (4) investigate the influence of Fe^2+^ on the expression of the catalase genes *katG* and *katE,* produced by *E. coli* strains.

## Methods

### Bacterial strains

Ten *E. coli* strains were isolated from the inflamed colonic mucosa of 10 adult patients in the acute phase of UC, which was confirmed by severe endoscopic changes in their colon. Severity of symptoms in the patients was also determined on the basis of the Mayo Clinic disease activity index [2]. Biopsies were obtained during colonoscopy procedures carried out at the Clinic of Gastroenterology of the Jagiellonian University Medical College in Krakow, Poland, after approval by the Jagiellonian University Bioethical Committee (no. KBET/75/B from 15.11.2007). Informed consent was obtained from all patients participating in the study.

All subjects underwent the same type of preparation prior to colonoscopy, with oral sodium phosphate at a dose of 0.6–0.8 ml/kg (up to 45 ml) and bowel cleansing, consisting of four saline enemas. During colonoscopy, patients received intravenous sedation or general anesthesia, as required. The biopsy samples were taken with sterile tools with extreme caution for sterility by staff. The samples were placed in sterile tubes, suspended in Schaedler broth (SAB; Difco, USA) with 10 % glycerol and stored at −20 °C for up to 1 week. Biopsy samples were transported to the laboratory Department of Microbiology, Jagiellonian University Medical College on dry ice.

Frozen samples were thawed and homogenized in 1 ml of Schaedler’s medium (Oxoid, Hampshire, UK). Different media were used to cultivate bacteria under aerobic and anaerobic conditions to analyze the samples quantitatively for the main bacterial constituents [14].

Methods of preparing samples and media used for cultivation of particular groups of bacteria, were described in our previous paper [15].

Phenotypic identification of *E.coli* isolates from McConkey Agar was conducted with the commercial identification system API20E (BioMerieux, Marcy l’Etoile, France). To confirm the species designation, all isolates of Gram-negative rods were tested using the PCR method with species-specific primers for *E. coli* [16]. Stock cultures of the isolated strains were preserved at -80 °C on glass beads in BBL nutrient broth with 15 % glycerol (BD). Ten *E. coli* strains randomly selected from our collection were included in the study (Table 1).

**Table 1:** List of *E. coli* strains (EC1-EC10) used in the experiment. Species identification was conducted with the API 20E system. PCR was used to confirm the presence of genes encoding catalases (*katG* and *katE*), a ferrous transporter (*feoB*), and receptor proteins for siderophores (*fepA, fhuA*, *fecA, iroN, fyuA,* and *iutA*)

*E. coli* strain number	Gene presence								
	*katE*	*katG*	*feoB*	*fepA*	*fhuA*	*fecA*	*iroN*	*fyuA*	*iutA*
EC1	1+	1+	1+	1+	1+	1+	1+	1+	1+
EC2	1+	1+	1+	1+	1+	1-	1+	1+	1+
EC3	1+	1+	1+	1+	1+	1-	1-	1-	1-
EC4	1+	1+	1+	1+	1+	1-	1-	1-	1-
EC5	1+	1+	1+	1+	1+	1-	1-	1-	1-
EC6	1+	1+	1+	1+	1+	1+	1+	1+	1-
EC7	1+	1+	1+	1+	1+	1+	1+	1+	1+
EC8	1+	1+	1+	1+	1+	1+	1-	1+	1+
EC9	1+	1+	1+	1+	1-	1-	1-	1-	1-
EC10	1+	1+	1+	1+	1+	1-	1-	1-	1-

### Detection of genes encoding catalases and iron acquisition proteins

PCR was used to detect: *katG* and *katE*, which encode enzymes that catalyze H_2_O_2_ decomposition; *feoB*, which encodes a Fe^2+^ transporter protein; *fepA*, *fhuA*, *fecA, iroN, fyuA*, *iutA*, which encode siderophore receptors. Primer sequences and amplification product sizes are shown in Table 2.

**Table 2:** Primers used in this study

Gene	Primers sequences	Product size [bp]
16S rRNA	5′-GGG AGT AAA GTT AAT ACC TTT GC-3′ 5′-CTC AAG CTT GCC AGT ATC AG-3′	204
*katE*	5′-AAC GAG TGA GGC TTT ACC TGC-3′ 5′-AAC CTG AAA CTC TGC ACA ACG-3′	173
*katG*	5′-CTG CGT TTT GAT CCT GAG TTC–3′ 5′-GGC CCG ATG TAG CGA GAT T-3′	137
*feoB*	5′-CGT GTA GGT AAC TGG GCT GGC-3′ 5′-AGG TCT GCG ATG AGA TGG TGG-3′	127
*fepA*	5′-AGC TGA CTG ACA GCA CCA TCG-3′ 5′-CGG GAT GAT CGA CAA ACG GTC G-3′	554
*fhuA*	5′-AGA CAC TAT CAC CGT TAC CGC TG-3′ 5′- GCC GCG AAT GAT CAG GTG GTC-3′	265
*fecA*	5′-AGG TTA ATA TCG CAC CGG GAT CG-3′ 5′-ATG GCA TCC ATG TTG CCG AGC-3′	565
*iroN*	5′-AAG TCA AAG CAG GGG TTG CCC G-3′ 5′-GAC GCC GAC ATT AAG ACG CAG-3′	667
*fyuA*	5′-GCA GTA GGC ACG ATG TTG TA-3′ 5′-TGA TTA ACC CCG CGA CGG GAA-3′	377
*iutA*	5′-GGC TGG ACA TCA TGG GAA CTG G-3′ 5′-CGT CGG GAA CGG GTA GAA TCG-3′	302

### Influence of Fe^2+^ on the growth rate of *E. coli*

The 10 *E. coli* strains were grown overnight at 37 °C in *McConkey* agar *(*Oxoid)*.* Each strain was inoculated in 10 ml of tryptic soy broth (TSB) (BD) and incubated at 37 *°*C for 3 h. After incubation, probes were microcentrifuged (5000 rpm, 10 min, 4 *°*C), supernatants were removed, and pellets were resuspended in 10 ml of phosphate buffered saline (PBS). This step was repeated three times*.* Subsequently, each sample was diluted 1:3 in PBS. Bacterial density in prepared samples was determined by measuring optical density (JASCO Corporation Spectra Manager v.1.30.01) at a wavelength of 600 nm (OD_600_) in triplicate. Inoculums of the *E. coli* strains had a similar OD reading 0.5 ± 0.02, (equivalent to 1 × 10^5^ colony forming units/ml).

From this culture, 100 μl of bacterial inoculum were added to 10 ml of one of four growth media: M9(-), M9(Fe^2+^), TSB, or PBS. Defined minimal medium (M9(-)) consisted of 15.2 g of (Na_2_HPO_4_)12H_2_O, 3 g of KH_2_PO_4_, 0.5 g of NaCl, 1 g of NH_4_Cl, 0.0015 g of (CaCl_2_)2H_2_O, 1 ml of 1 M (MgSO_4_)7H_2_O, 1 ml of 0.1 M CaCl_2_, 10 ml of 20 % glucose, and 1000 ml of H_2_O. To remove iron from the medium, Chelex 100 (BioRad, Hercules, USA) was used in accordance with the manufacturer’s instructions. Defined minimal medium supplemented with ferrous ions (M9(Fe^2+^)) consisted of M9 minimal medium with 0.2 mM (FeSO_4_)7H_2_O, stabilized with 0.3 mM EDTA. TSB was used as the positive, while PBS as the negative control.

To follow bacterial growth, the OD_600_ was measured every 2 h for 12 h. The last measurement was made after 24 h of incubation at 37 °C.

### Impact of Fe^2+^ on the kinetics of H_2_O_2_ decomposition by *E. coli*

The bacterial strains were cultured in 10 ml of M9(-), M9(Fe^2+^), TSB, or PBS for 24 h at 37 °C. Probes were microcentrifuged (5000 rpm, 10 min, 4 *°*C) and diluted in 10 ml of PBS to a final OD_600_ of 0.5 ± 0.02, (equivalent to 1 × 10^5^ colony forming units/ml). Then, 100 μl of the diluted bacterial culture were added to 10 ml of M9(-), M9(Fe^2+^), TSB, or PBS. Immediately afterward, chemically pure H_2_O_2_ (Sigma-Aldrich) was added to each culture for a final concentration of 60 mg/L. The culture was incubated at 37 °C. The amount of H_2_O_2_ remaining in the test tube was determined every 10 min by using Analytical Merckoquant peroxide test strips (Merck, NJ, USA). Negative controls for each media type with added H_2_O_2_ and no *E. coli* were included.

### Catalase gene expression change in response to Fe^2+^

The 10 *E. coli* strains were grown in M9(-), M9(Fe^2+^) or TSB media, as described above. Cells were collected from two time points at an OD_600_ of approximately 0.6, 1.0 for M9(-), 0.6, 1.0 for M9(Fe^2+^) and 0.6, 2.0 for TSB. Total RNA was extracted with the RNeasy Mini Kit (Qiagen, Venlo, Holand), according to the manufacturer’s instructions. RNA was diluted in diethylpyrocarbonate treated water (A&A Biotechnology, Gdynia, Poland), and 100 ng aliquots were treated with RNase-free DNase (Sigma-Aldrich), in accordance with the manufacturer’s instructions. Following treatment, 20 ng of RNA were converted to cDNA using the M-MuLV reverse transcriptase (RT) synthesis system (Thermo Scientific, Waltham, MA, USA). Random hexamers supplied with the kit were used to initiate cDNA synthesis.

RT-PCR was carried out using a Real-Time PCR CFX96 thermal cycler (BioRad). Reactions were carried out with the SYBR Green PCR Master Mix (Sigma-Aldrich). Cycling parameters were optimized to ensure primer efficiency. Cycling parameters for *katG* and *katE* primers were 95 °C for 30 s, 60 °C for 1 min, and 72 °C for 45 s. All reactions were performed in triplicate; 16S expression was used to normalize the results. To confirm that only products of interest were formed, the melting curve and product sizes of each reaction were analyzed. Relative levels of gene expression were calculated by the 2^−ΔΔCt^ (comparative Ct) method [17]. The 16S gene was chosen as the loading control.

### Statistical analysis

Statistical analysis was carried out using software packages: Access and Statistica.

Likelihood ratio and *χ*2 tests were used. A *p*-value < 0.05 was considered statistically significant.

## Results

### Occurrence of genes encoding catalases (*katG, katE*) and iron acquisition proteins (*feoB, fepA, fhuA, fecA, iroN, fyuA, iutA*)

Using PCR, we examined the occurrence of genes encoding catalases and iron acquisition proteins in the 10 *E. coli* strains isolated from inflammatory sites of intestinal tissues from UC patients. A gene for a Fe^2+^ transporter protein (*feoB*), which contributes to ferrous acquisition, and genes encoding enzymes that catalyze H_2_O_2_ decomposition (*katG* and *katE*) were present in all investigated strains. Each *E. coli* strain had at least one gene encoding a siderophore receptor (*fepA*, *fhuA*, *fecA*, *iroN*, *fyuA*, *iutA*). The results are shown in Table 1.

### Influence of ferrous ions on *E. coli* growth rate

**Fig. 1:**
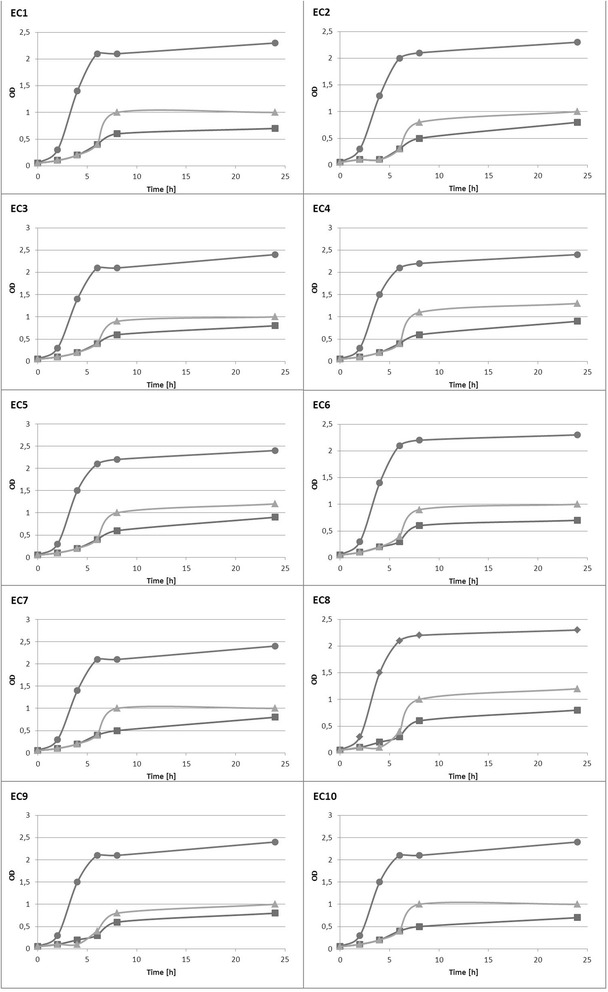
Bacterial growth curves for the 10 investigated *E. coli* strains (EC1-EC10) in TSB media (circle), M9(Fe^2+^) media (triangle), and M9(-) media without iron ions (square)

We used OD_600_ measurements to detect whether *E. coli* grew differently in the presence or absence of Fe^2+^ in the compared media. All investigated strains of *E. coli* grew fastest in TSB (Fig. 1), which contains all of the necessary nutritive elements for growth and was included as a positive control. The presence of ferrous ions in the M9(Fe^2+^) medium increased the *E. coli* growth rate compared to the M9(-) medium. Statistical analysis, profiled by quadratic equation (R2 = 0,93; f = 1196,267; *p* < 0,0001), confirmed that all 10 strains, *E. coli* cultured in M9(Fe^2+^) reached the log phase of growth faster and had higher OD_600_ values after incubation for 24 h compared to *E. coli* cultured in M9(-).

### Influence of ferrous ions on kinetics of H_2_O_2_ decomposition by *E. coli*

**Fig. 2:**
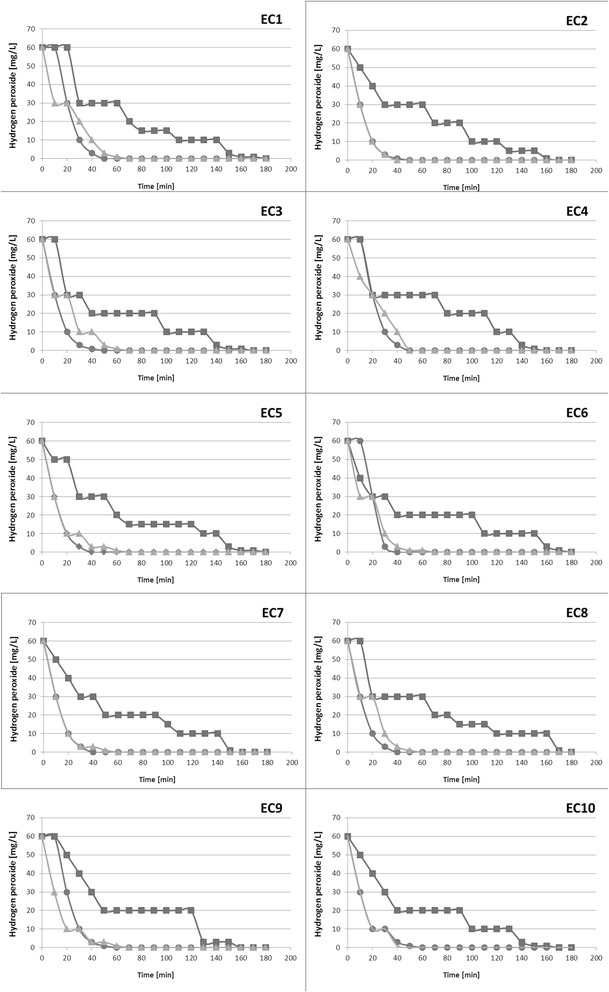
Catalase activity for the 10 *E. coli* strains (EC1-EC10) in in TSB media (circle), M9(Fe^2+^) media (triangle), and M9(-) media without iron ions (sqare)

To understand the impact of ferrous ions on the activity of catalases produced by *E. coli,* we monitored the kinetics of H_2_O_2_ decomposition (Fig. 2). We observed statistically significant differences in the ability to convert H_2_O_2_ between strains cultured in M9(-) and M9(Fe^2+^) media. *E. coli* strains incubated in M9(Fe^2+^) decomposed H_2_O_2_ faster than strains incubated in M9(-) (f = 165.9024, *p* < 0.0001). These results indicate that the presence of Fe^2+^ in growth media influences catalase activity.

### *KatE and katG* gene expression is upregulated in the presence of ferrous ions in growth media

**Fig. 3:**
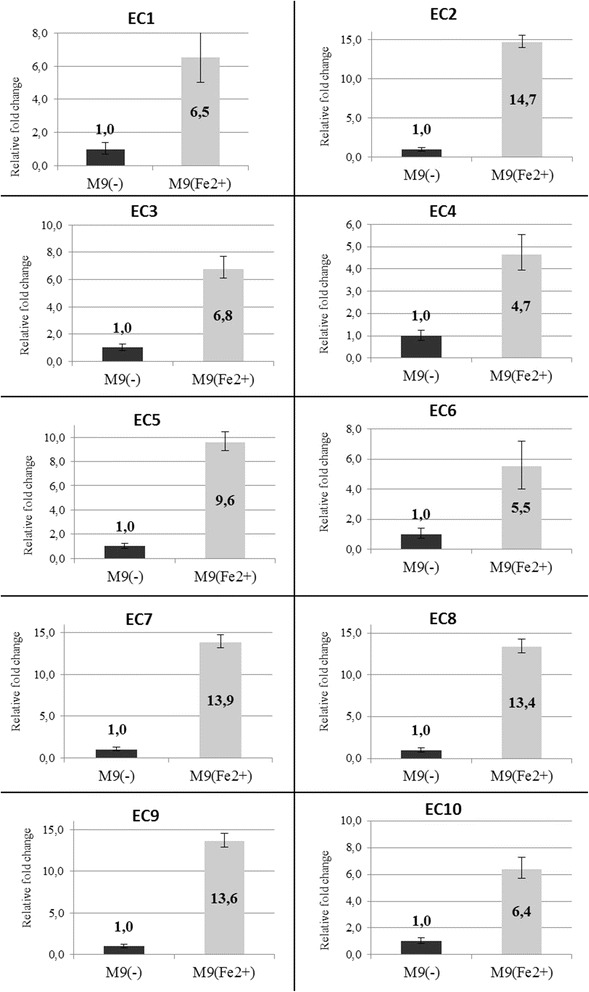
Relative expression of *katG* gene in the 10 investigated *E. coli* strains (EC1-EC10). Fold change in gene expression is presented for *E. coli* strains cultured in M9(Fe^2+^) media (grey bars) compared to strains incubated in M9(-) media (black bars). Expression was measured during logarithmic phase of bacterial growth by quantitative real-time PCR

We used quantitative real-time PCR to examine catalase (*katE* and *katG*) genes expression in response to the presence of Fe^2+^ in growth media. The 16S gene was used as a reference to analyze the relative changes in *katG* and *katE* expression. The *katG* expression was upregulated in all investigated *E. coli* strains cultured in M9(Fe^2+^) compared to *E. coli* cultured in M9(-) in the logarithmic and stationary phases of bacterial growth (Fig. 3). However, *katE* expression was not dependent on the presence of iron ions.

## Discussion

Increased knowledge of UC pathophysiology requires better understanding of how interactions among bacterial factors, host genetics, and the immune system result in chronic mucosal inflammation [3]. As the largest population of microbes in the human body, the gut microbiota, has been implicated in many aspects of UC [5, 18]. Members of the *Enterobacteriaceae* family, especially *E. coli,* may play important roles in the pathogenesis of inflammatory bowel disease (IBD)*.* There is substantial controversy regarding the abundance of *E. coli* in the colonic mucosa of UC patients. Earlier studies have demonstrated that patients with UC or Crohn disease have increased *E. coli* populations in the gut [19]. In our previous study on populations of *E.coli* present in colon of the UC patients we also noticed that as to compared to the control group, there was a significant increase in the number of *E. coli* bacteria at the site of inflammation in patients with UC [15]. On the other hand, several works have consistently reported no increase of *E.coli* numbers in UC patients with respect to healthy subjects [20]. Discrepancies may be explained by differences in the disease severity of the patients included to these studies and activity status in UC patients among study groups. Thus, the increase in numbers of *E. coli* may depend on different factors. Biofilm formation and the abilities to absorb ions and nutrients from the environment, but also on their abilities to breakdown ROS in the specific niche like as inflammatory site seem to most important [11, 21]. Thus, the question arises: what is the role of *E. coli* population in course of UC? The standard opinion is that *E. coli* may contribute to the propagation of the chronic inflammation in UC by activating the local immune system. However, on the other hand, the increased numbers of *E. coli* in consequence intensify iron acquisition and catalase activity and on that way may prevent the Fenton reaction and, thus, induce remission of UC.

In this work, we aimed to identify pathways on which *E. coli* may be involved in the induction and/or maintenance of UC remissions. Hypotheses about the effect of probiotic *E. coli* strain on the health of patients in the acute phase of UC by inducing remission of the inflammatory process was raised by Seksik [22].

In our previous studies we have not only quantitatively assessed *E. coli* in UC patients as against to a control group, but also analyzed the genetic traits of *E. coli*. We performed comparative analysis of 52 *E. coli* strains, using Pulsed-Field Gel Electrophoresis. On the basis of these results was selected 10 strains of *E. coli*. Described strains were isolated from 10 different patients with acute phase of UC and characterized by different pulsotypes. Analysis of 52 restriction patterns revealed high great genetic variability diversity among *E. coli* strains isolated from inflamed mucosa. Moreover, the local inflammation status was not related to colonization by a specific type of *E. coli* [19]. Thomazini et al. obtained similar results using enterobacterial repetitive intragenic consensus-PCR in an analysis of 131 *E. coli* strains isolated from IBD patients (including patients with UC) and a control group. No specific strain or group of *E. coli* strains correlated with UC, Crohn disease, or the control group [23]. Earlier, Sepehri and colleagues using multilocus sequence typing, made a comparison of *E. coli* strains isolated from IBD patients (including UC) with those from the control group. They identified three main groups of the bacteria*,* but did not find any relationship between strains and disease type [24]. Thus, as it has been demonstrated by several authors and us, no particular strain or group of *E. coli* strains is associated with UC or Crohn disease.

Inflammatory sites in the colon mucosa contain large amounts of H_2_O_2_ from the immune cell-initiated explosion of oxygen and high concentrations of Fe^2+^ from degraded heme molecules. Both H_2_O_2_ and Fe^2+^ are substrates for the Fenton reaction, which produces the most active of all oxygen forms, the hydroxyl radicals [25]. Hydroxyl radicals can react with almost all substances and can cause the peroxidation of lipids, the disruption of cell membranes, and the inhibition of enzymes that transport Ca^2+^ and Mg^2+^ ions across membranes [9, 11, 26]. For most microbes, iron is an essential element involved in multiple metabolic processes, including respiration and critical enzymatic reactions. It is difficult for bacteria that colonize the human GI tract, such as *E. coli*, to obtain enough iron for their metabolism. Therefore, bacteria have developed several systems to allow the absorption of Fe^2+^ and Fe^3+^, as well as to acquire iron by destruction of hemoglobin [11, 12]. We sought to determine whether the number of genes that regulate iron acquisition in *E. coli* (*fepA*, *fhuA*, *fecA*, *iroN*, *fyuA*, and *iutA*) influences the growth rate of a bacterial population. These genes are involved in the biosynthesis of transporter proteins for several siderophores [11]. Regardless of the number of iron acquisition genes in their genomes, all *E. coli* strains grew significantly faster in the presence than in the absence of Fe^2+^. Thus, it appeared that any product of each of the detected genes may compensate for the absence of other proteins. Notably, all of the tested *E. coli* strains had the *feoB* gene, which biosynthesizes a protein that directly transports Fe^2+^ into the periplasm. Therefore, at inflammatory sites, *E. coli* may act as a specific chelator, or iron ion catcher, of both the second and third oxidation states of iron. It has been demonstrated that an excess of the soluble iron given orally has been related to deleterious changes to the gut microbiota in gastrointestinal diseases. and with increased growth and adherence of the enteric pathogens [27], while specific iron chelators have been proposed to inhibit the Fenton reaction and to improve the health status of patients with UC [25]. More recently, Werner and colleagues [28] demonstrated that depletion of iron in the gut prevented development of chronic inflammation in a murine model of Crohn’s disease. It may be possible to use *E. coli* in a similar way as an iron-chelating agent in chronic inflammation.

In the acute phase of UC, the activity of an increased number of ROS intensifies the necrotic processes of the intestinal epithelium. *E. coli* may eliminate Fenton reaction substrates by biosynthesizing enzymes that catalyze H_2_O_2_ decomposition to oxygen and water. *E. coli* produces two inducible hydroperoxidases, HPI and HPII, which differ in structure and kinetic activity [29]. We tested whether *E. coli* strains isolated from patients in the acute phase of UC produced these catalases. All *E. coli* strains had both the HPI-encoding *katG* gene and the HPII-encoding *katE* gene. When cultured in TSB media, all strains were able to decompose H_2_O_2_. The presence of Fe^2+^ in the culture media accelerated H_2_O_2_ decomposition by the *E. coli* strains compared to the culture medium without iron supplementation. Accordingly, we hypothesize that *E. coli* strains that cannot biosynthesize the active form of catalases would be unable to colonize the gastrointestinal tract of patients in the acute phase of UC because the oxidative stress level would be too high for them.

**Fig. 4:**
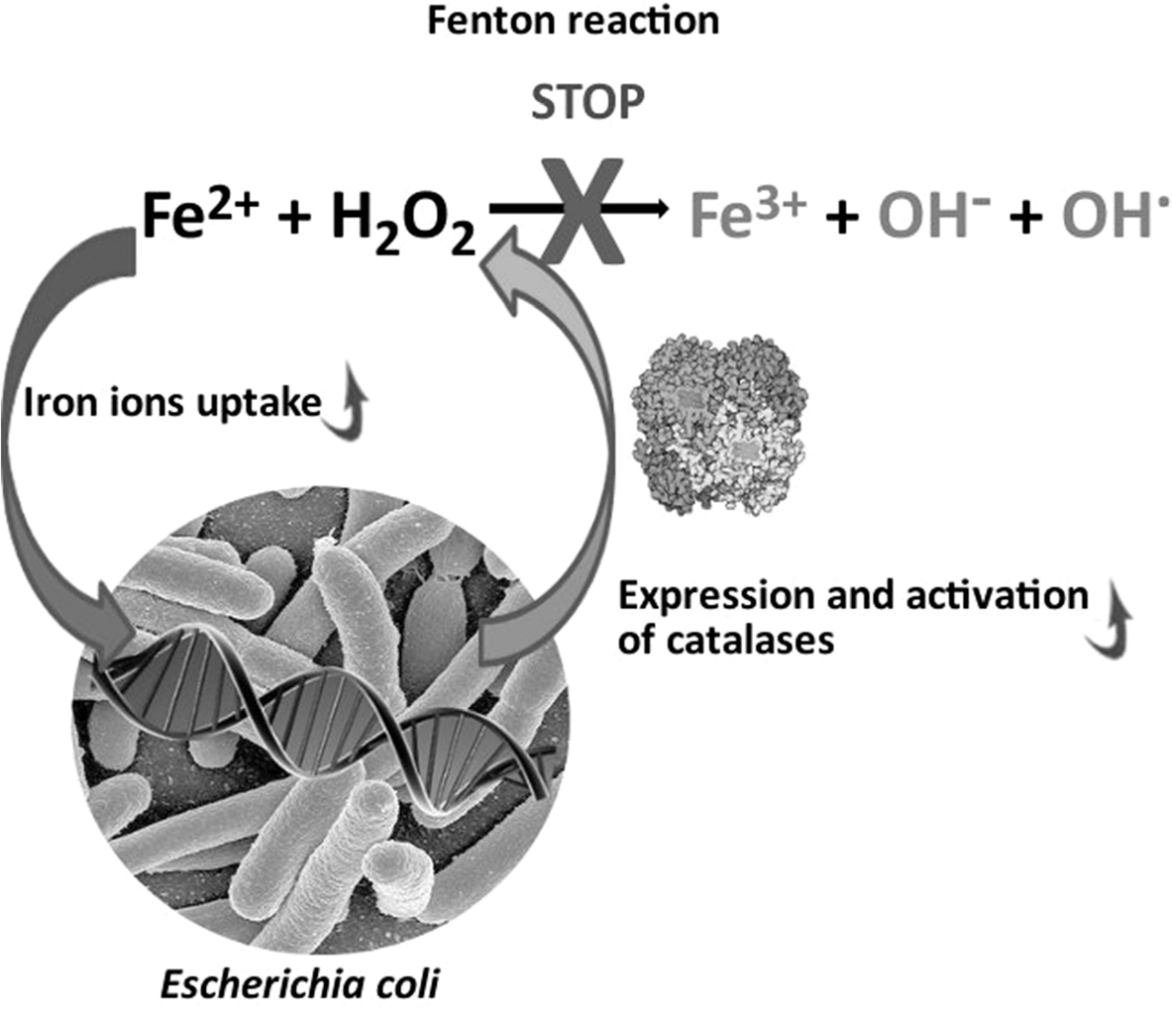
Role of *E. coli* in inhibiting the Fenton’s reaction

Next, we determined the *katG* and *katE* gene expression levels in response to the presence of elevated Fe^2+^ concentrations in the media. Real-time PCR revealed that Fe^2+^ induces *katG* gene expression. In all tested *E. coli* strains, in the logarithmic and stationary growth phases, the *katG* expression level was highest in media with Fe^2+^. Notably, we observed a 15-fold increase in *katG* gene expression for one *E. coli* strain incubated in Fe^2+^-enriched media as compared to M9(-). However, the presence of Fe^2+^ in the media did not affect the expression of *katE* in any of the tested *E. coli* strains. Literature data [13] indicate that gene expression *katE* depends on the growth stage of the the bacteria culture and is much higher during the stationary phase. In the acute phase of UC population of *E.coli* are predominantly maintained in logarithmic growth phase. On the base of our results, we can hypothesize that *E. coli* populations may participate in the rapid elimination of Fe^2+^ at sites of active inflammation. With an increase in the *E. coli* numbers, there would be an increased biosynthesis of enzymes catalyzing H_2_O_2_ and its decomposition to water and oxygen. In this way, *E. coli* may eliminate the two substrates of the Fenton reaction and, thus, induce the initiation or prolongation of remission stages of UC (Fig. 4). It is of interest that long before contemporary studies on a possible role for *E. coli* in ameliorating clinical course of IBD, researchers had proposed the use of *E. coli* strain Nissle 1917 as a probiotic to improve the course of the disease. Clinical effects of using this strain has been reported and it is considered to be the equivalent of mesalazine, a drug used to maintain remissions in UC patients [6, 30]. It is, therefore, possible that one of the mechanisms of the inhibition of the inflammatory reactions in UC underlying natural or intentional presence of high *E.coli* numbers in the intestine of UC patients is related to inhibition of the Fenton reaction. Thus, it seems that there is a possibility that nonpathogenic *E. coli* strains will be used successfully as probiotics to induce and perpetuate remissions in UC. Such a hypothesis should be verified in a well-designed, long-term clinical studies in which the same patients receiving selected *E.coli* strain would be observed during active and quiescent phases of the disease in comparison to control subjects.

It should be noted, that presented experiments are beginning research on the role of microbiota in inflammatory processes associated with UC. In consideration should be taken role of other groups of bacteria mainly *Enterococcus* in the course of inflammatory bowel disease. Our study was also limited by a low numer of the samples taken from the eligible patients. Moreover, unfortunately, it was not possible to measure concentrations of iron ions and hydrogen peroxide at the site of inflammation during surgery, or colonoscopy. To better understand the conditions occurring at the site of inflammation, we should taking into account the proportion of cells of the immune system. This will provide a better model for in vitro studies.

## Conclusions

More attention is devoted to the role of the *E. coli* in the inflammatory processes accompanying UC. On one hand, the numbers of the *E. coli* are significantly increased in inflamed colon, which may lead to the activation of the cells of the immunological system, whilst, on the other hand, the *E. coli* present the features which might affect the resolution of the inflammatory reactions in the intestines. In the acute phase of UC, necrotic processes of the intestinal epithelium are intensified as a result of the action of an increased number of reactive oxygen species. The *E. coli* may eliminate the substrates of Fenton’s reaction by means of assimilating ferrous (Fe^2+^) and by the biosynthesis of the enzymes catalysing the decomposition of oxygen peroxide. In this way, the *E. coli* may inhibit the formation of hydroxyl radicals, by which they may affect the initiation and/or prolongation of remission stages of UC.

## Abbreviations

GI: Gastrointestinal tract
HPI and HPII: Hydroperoxidases
IBD: Inflammatory bowel diseases
PCR: Polymerase chain reaction
ROS: Reactive oxygen species
UC: Ulcerative colitis
